# Associations of Coping Strategies With Glycemic and Psychosocial Outcomes Among Adolescents With Type 1 Diabetes Experiencing Diabetes Distress

**DOI:** 10.1093/abm/kaae028

**Published:** 2024-06-03

**Authors:** Emma Straton, Kashope Anifowoshe, Hailey Moore, Randi Streisand, Sarah S Jaser

**Affiliations:** Children’s National Hospital Division of Psychology and Behavioral Health, Washington, DC, USA; Vanderbilt University Medical Center Department of Pediatrics, Nashville, TN, USA; Children’s National Hospital Division of Psychology and Behavioral Health, Washington, DC, USA; Children’s National Hospital Division of Psychology and Behavioral Health, Washington, DC, USA; The George Washington University School of Medicine Department of Psychiatry and Behavioral Sciences, Washington, DC, USA; Vanderbilt University Medical Center Department of Pediatrics, Nashville, TN, USA

**Keywords:** Adolescents, Type 1 diabetes, Coping, Quality of life

## Abstract

**Background:**

Many adolescents with type 1 diabetes experience diabetes distress which is associated with suboptimal glycemic and psychosocial outcomes. The ways in which adolescents respond to diabetes distress may serve as a risk or protective factor for these outcomes, but few studies have examined the coping strategies adolescents use to manage diabetes distress.

**Purpose:**

To examine the association of coping strategies with glycemic and psychosocial outcomes among adolescents experiencing diabetes distress.

**Methods:**

Participants included 198 adolescents with elevated diabetes distress who completed baseline data for a randomized controlled trial (*M*_age_ = 15.3 ± 1.4, 58% female, 58% non-Hispanic White, *M*_A1c_ = 9.1 ± 2.1%). Adolescents reported on their use of coping strategies related to diabetes stressors, including primary control engagement coping (e.g., problem-solving), secondary control engagement coping (e.g., positive thinking), and disengagement coping (e.g., avoidance). Adolescents also completed measures of diabetes distress, quality of life, and resilience. HbA1c data were extracted from electronic medical records and at-home kits.

**Results:**

Higher use of primary control engagement coping was associated with better glycemic and psychosocial outcomes. Secondary control engagement coping was associated with better psychosocial outcomes but not glycemic outcomes. Greater use of disengagement coping strategies was associated with poorer glycemic and psychosocial outcomes. All associations were significant after adjusting for adolescent sex, age, race/ethnicity, and continuous glucose monitor use.

**Conclusions:**

These results build on prior findings by including a more diverse sample of adolescents and highlight the value of promoting engagement coping strategies and discouraging the use of disengagement coping strategies among adolescents experiencing diabetes distress.

**Clinical Trial information:**

NCT03845465.

## Teaser

Among a diverse group of adolescents with type 1 diabetes experiencing diabetes distress, engagement coping strategies, (e.g., problem solving), were associated with better glycemic and psychosocial outcomes, while disengagement strategies, (e.g., avoidance), were associated with worse outcomes

## Introduction

Type 1 diabetes (T1D) is one of the most common chronic diseases in childhood, and the incidence of T1D onset has been increasing in adolescents [1]. Furthermore, adolescents from minoritized racial and ethnic groups are diagnosed with T1D at a steeper rate than non-Hispanic White youth [2]. Adolescents in the general population commonly experience elevated stress due to developmental changes, an increased desire for independence, and a greater emphasis on the development and maintenance of peer relationships [3]. Youth with T1D face additional stressors during adolescence including, but not limited to, learning to independently manage their T1D regimen (e.g., monitoring blood glucose levels, carbohydrate counting, and administering insulin) and perceived stigma related to having diabetes [4]. Previous research indicates that increased diabetes-related stress can contribute to negative impacts on T1D management [5]. Furthermore, only 18% of adolescents with T1D meet the American Diabetes Association (ADA) target HbA1c of <7.0% which is an indicator of risk for diabetes complications [6].

Diabetes distress, or the negative emotions related to the burden of managing diabetes, is prevalent among adolescents with T1D, with elevated levels reported by about one-third of adolescents, and even higher rates among youth from minoritized racial and ethnic groups [7–9]. Diabetes distress is not only associated with emotional challenges but also has associations with glycemic outcomes; higher levels of diabetes distress have consistently been associated with higher HbA1c [10]. As diabetes distress symptoms intensify, so does the challenge of maintaining optimal blood glucose levels, leading to a greater risk of diabetes-related complications (e.g., severe hypoglycemic events or diabetic ketoacidosis) [11]. Furthermore, diabetes distress has been correlated with lower health-related quality of life (HRQOL) for adolescents with T1D [12].

Chronic stress is known to lead to negative physical and mental health outcomes [13]. The burden of diabetes management poses the potential for chronic stress in adolescents with T1D; however, not all adolescents who experience these stressors develop negative glycemic and psychosocial outcomes. Many adolescents with T1D demonstrate resilience in response to the stressors related to T1D management [14, 15]. The ways adolescents respond to stress may serve as risk or protective factors for negative glycemic and psychosocial outcomes.

Coping strategies, or the controlled and volitional responses to stress, are often defined using a control-based model [15, 16]. This model includes primary control engagement coping (i.e., acting on the source of a stressor, which may include problem-solving or emotional expression), secondary control engagement coping (i.e., adapting to a stressor, which may include acceptance or cognitive reappraisal), and disengagement coping (i.e., disengaging from a stressor source, including avoidance or denial) [15]. In the general population, the use of primary and secondary control coping strategies is related to lower levels of internalizing and externalizing symptoms in adolescents, while the use of disengagement coping strategies is related to higher levels of psychopathology among children and adolescents [15]. Less is known about which strategies may be most adaptive for responding to diabetes-related stressors, which are often uncontrollable.

Previous work with adolescents with T1D demonstrated that adolescents’ use of primary and secondary control engagement coping predicted higher quality of life and fewer depressive symptoms over 12 months, while the use of disengagement coping strategies predicted lower quality of life and more depressive symptoms [12]. Coping strategies were not associated with glycemic outcomes [12]. Yet, the sample included in this previous study was nearly three quarters non-Hispanic White with a relatively low average HbA1c of 7.5% and may not be generalizable to the population of adolescents with T1D [12]. In the current study, we build on this work by using data from a larger, more diverse sample, along with additional measures of resilience and diabetes distress.

Based on earlier studies [12, 15], we hypothesized that higher use of primary control engagement coping strategies would be associated with more positive outcomes including lower HbA1c, less diabetes distress, and higher HRQOL; higher use of secondary control engagement coping strategies would be associated with less diabetes distress, higher HRQOL, but not HbA1c since active problem-solving may be needed to cope with stressors related to diabetes management tasks; and higher use of disengagement coping strategies would be associated with higher HbA1c, more diabetes distress, and lower HRQOL. Additionally, we expected adolescents who demonstrated higher resilience to also report higher use of primary control coping strategies, such as problem-solving [17].

## Methods

The current study is a secondary analysis of baseline data from adolescents enrolled in a multisite randomized controlled trial examining the efficacy of a positive psychology intervention to treat diabetes distress in adolescents with T1D [18]. Adolescents were eligible if they were aged 13–17 years, ≥1 year since T1D diagnosis, fluent in English, experiencing at least moderate diabetes distress (total score ≥34) using the Problem Areas in Diabetes-Teen (PAID-T) scale [19], and had a text-messaging capable device. Exclusion criteria included other serious health conditions that interfere with diabetes care. Informed assent and consent were obtained from all adolescent and caregiver participants included in the study. Baseline data were collected from December 2019 to July 2022.

Coping strategies used by adolescents in response to diabetes-related stressors were assessed using the Responses to Stress Questionnaire, Type 1 Diabetes Version (RSQ) [16]. The first 10 items assess diabetes-related stressors commonly experienced by adolescents over the past 6 months and yield a Total Stress Score ranging from 0 to 30, with higher scores indicating greater diabetes-related stress. The next 57 items ask adolescents to indicate how they respond to these stressors. Three coping factors were used in the analysis: primary control engagement coping (e.g., problem-solving or emotional expression), secondary control engagement coping (e.g., acceptance or cognitive reappraisal), and disengagement coping (e.g., avoidance or denial). Ratio scores were used to indicate the relative use of strategies. Cronbach’s alpha for this sample was 0.72 for primary control engagement coping, 0.72 for secondary control engagement coping, and 0.81 for disengagement coping.

To assess diabetes distress, adolescents completed the PAID-T, a measure validated for use in adolescents with T1D. PAID-T scores range from 14 to 84, with scores ≥44 considered clinically significant. Cronbach’s alpha for this sample was 0.95 [19]. Diabetes-specific HRQOL was assessed using the Type 1 Diabetes and Life (T1DAL) measure, a validated 23-item measure with scores ranging from 0 to 100 and higher scores indicating better HRQOL [20]. Cronbach’s alpha for this sample was 0.89. The Diabetes Strengths and Resilience Measure for Adolescents (DSTAR-Teen) [17], a validated 12-item measure with scores ranging from 12 to 60, was used to measure adolescent resilience related to diabetes management [17]. Cronbach’s alpha for this sample was 0.89. Caregivers reported on their adolescents’ demographic information. HbA1c data were extracted from electronic medical records (point-of-care values) and at-home HbA1c kits (for adolescents who had telehealth diabetes visits).

Baseline data were collected between 2019 and 2022. All measures met skew and kurtosis normality assumptions. Descriptive statistics were used to summarize sample demographic, medical, and psychosocial characteristics. Variation in coping strategy usage by demographic and medical characteristics was examined using analysis of variance, independent samples *t* tests, and Pearson’s *r* bivariate correlations. Pearson’s *r* bivariate correlations were performed to examine the association between coping strategies usage with HbA1c, diabetes distress, HRQOL, and diabetes-related resilience. Linear regression models were fitted to assess the associations of coping strategies with the glycemic and psychosocial measures adjusting for preselected covariates (adolescent sex, age, race/ethnicity, and continuous glucose monitor use). All analyses were performed using IBM SPSS Statistics software, v28.

## Results

Participants included 198 adolescents who endorsed elevated diabetes distress (see Table 1 for demographic, medical, and psychosocial characteristics). HbA1c values were collected at baseline from 94% of participants, and only 14% of participants met the ADA target HbA1c of <7.0% [21]. Additionally, 62% of study participants met the established clinical cutoff for diabetes distress with a PAID-T score of ≥44 [19].

**Table 1:** Demographic, Diabetes, and Psychosocial Participant Characteristics

Demographic characteristics	Adolescent age, years (mean [*SD*])	15.3 [1.4]
	Adolescent sex, female (*N* [%])	115 [58%]
	Adolescent race (*N* [%])	
	White	123 [62%]
	Black/African American	47 [24%]
	Asian	8 [4%]
	American Indian or Alaska Native	2 [1%]
	Biracial	16 [8%]
	Another race	2 [1%]
	Adolescent ethnicity (*N* [%])	
	Non-Hispanic or Latinx	189 [96%]
	Hispanic or Latinx	9 [4%]
Diabetes factors	T1D duration, years (mean [*SD*])	6.3 [3.7]
	HbA1c, % (mean [*SD*])	9.1 [2.1]
	Continuous glucose monitor use (*N* [%])	161 [81%]
Psychosocial factors	PAID-T (mean [*SD*])	48.4 [10.5]
	T1DAL (mean [*SD*])	49.5 [12.6]
	DSTAR (mean [*SD*])	42.4 [7.4]
Coping strategies	Primary control coping (mean [*SD*])	0.15 [0.04]
	Secondary control coping (mean [*SD*])	0.23 [0.05]
	Disengagement coping (mean [*SD*])	0.16 [0.03]

*DSTAR* Diabetes Strengths and Resilience Measure for Adolescents; *PAID-T* Problem Areas in Diabetes-Teen; *T1D* type 1 diabetes; *T1DAL* Type 1 Diabetes and Life.

Use of primary and secondary control coping strategies varied significantly by adolescent sex, with male participants (*M*_primary control_ = 0.16, *M*_secondary control_ = 0.24) reporting higher use of primary and secondary control coping strategies than female participants (*M*_primary control_ = 0.15, *M*_secondary control_ = 0.23, *p* < .05). There was no significant difference in the use of disengagement strategies related to sex. The use of disengagement coping strategies varied significantly by adolescent race/ethnicity (*F* = 5.79, *p* = .004). Post hoc independent samples *t* tests revealed that non-Hispanic Black participants (*M*_disengagement coping_ = 0.17) demonstrated significantly higher use of disengagement coping strategies than both non-Hispanic White participants (*M*_disengagement coping_ = 0.16) and participants of another race or ethnicity (*M*_disengagement coping_ = 0.15, *p* < .005). There was no significant difference in primary or secondary control coping related to race or ethnicity. Finally, there was no significant difference in any coping strategy usage related to adolescent age, T1D diagnosis duration, or continuous glucose monitor use.

### Coping in Relation to HbA1c

As seen in Table 2, bivariate correlations revealed that greater use of primary control engagement coping strategies was associated with lower HbA1c while disengagement coping strategies were associated with higher HbA1c. HbA1c was not significantly associated with greater use of secondary control engagement coping strategies. After adjusting for preselected covariates (adolescent sex, age, race/ethnicity, and continuous glucose monitor use), primary control engagement coping strategy use was significantly associated with HbA1c (−13.9, 95% confidence interval [CI] [−22.1, −5.76], *p* < .001) as was disengagement coping strategy use (13.4, 95% CI [3.30, 23.5], *p* = .010). Secondary control coping strategy use remained unassociated with HbA1c (−4.90, 95% CI [−11.0, 1.22], *p* = .116). Each regression coefficient reported is the unstandardized beta.

**Table 2:** Bivariate Correlations Between Adolescent Coping Strategies and Glycemic and Psychosocial Outcomes

Variable	1	2	3	4	5	6	7
1. Primary control coping	–						
2. Secondary control coping	.53^***^	–					
3. Disengagement coping	−.55^***^	−.53^***^	–				
4. PAID-T	−.31^**^	−.31^***^	.31^***^	–			
5. T1DAL	.58^**^	.58^***^	−.51^***^	−.52^***^	–		
6. DSTAR	.45^**^	.27^***^	.41^***^	−.38^***^	.49^***^	–	
7. HbA1c	−.20^**^	−.08	.16^*^	.18^*^	−.15^*^	−.21^**^	–

*DSTAR* Diabetes Strengths and Resilience Measure for Adolescents; *PAID-T* Problem Areas in Diabetes-Teen; *T1DAL* Type 1 Diabetes and Life.

^*^ *p* < .05.

^**^ *p* < .01.

^***^ *p* < .001.

### Coping in Relation to Psychosocial Outcomes

Bivariate correlations revealed greater use of primary control engagement and secondary control engagement coping strategies was associated with fewer symptoms of diabetes distress, while disengagement coping strategies were associated with more symptoms of diabetes distress (Table 2). After adjusting for covariates (adolescent sex, age, race/ethnicity, and continuous glucose monitor use), primary control (−93.7, 95% CI [−134, −53.6], *p* < .001) and secondary control (−69.0, 95% CI [−98.1, −39.9], *p* < .001) engagement coping strategy use was still associated with fewer symptoms of diabetes distress, and disengagement coping strategy use was associated with more symptoms of diabetes distress (113.9, 95% CI [65.3, 163], *p* < .001).

Similarly, bivariate correlations showed that greater use of primary control and secondary control engagement coping strategies was associated with higher HRQOL. Disengagement coping strategies were associated with lower HRQOL (Table 2). After adjusting for covariates, primary control (200, 95% CI [159, 241], *p* < .001) and secondary control (143, 95% CI [113, 173], *p* < .001) engagement coping strategy use was significantly associated with higher HRQOL, and disengagement coping strategy use was associated with lower HRQOL (−212, 95% CI [−265, −160], *p* < .001).

Finally, bivariate correlations showed that greater use of primary control and secondary control engagement coping strategies was associated with higher diabetes-specific resilience. Disengagement coping strategies were associated with less resilience (Table 2). After adjusting for covariates, primary control (94.3, 95% CI [67.2, 121], *p* < .001) and secondary control (40.2, 95% CI [19.2, 61.1], *p* < .001) engagement coping strategy use was still associated with higher resilience, and disengagement coping strategy use was associated with lower resilience (−105, 95% CI [−138, −71.9], *p* < .001).

## Discussion

The current study is one of the first to examine the ways in which adolescents with T1D cope with diabetes-related stress in relation to HbA1c, diabetes distress, HRQOL, and resilience. We found that adolescents’ use of primary control engagement coping strategies, such as problem-solving or seeking social support, was associated with better glycemic and psychosocial (diabetes distress, HRQOL, and resilience) outcomes, even after adjusting for adolescent sex, age, race/ethnicity, and continuous glucose monitor use. Secondary control engagement coping strategy use, such as distraction or acceptance, was also associated with better psychosocial outcomes, but not with glycemic outcomes. Finally, disengagement coping strategy use, such as avoidance or denial, was consistently associated with worse glycemic and psychosocial outcomes.

The current study builds on previous work from a longitudinal study showing that coping strategies predicted quality of life and depressive symptoms in adolescents with T1D, but this earlier study had a less diverse sample (74% were non-Hispanic White) and more adolescents were meeting glycemic targets (mean HbA1c was 7.6%) [12]. The current study benefits from a larger, more diverse sample, and associations of coping strategies with HRQOL are consistent with this previous research. Additionally, we examined associations of coping strategies with diabetes distress which demonstrated similar associations as depressive symptoms in previous research [12]. The relationship between coping strategies and resilience is also consistent with the current literature [22, 23], yet our study was one of the first to examine these associations using a measure of resilience specifically designed for youth with diabetes (D-STAR [17]). Finally, the significant associations between coping and glycemic outcomes observed in our sample differ from the null findings previously reported, which may be due to the greater variability in HbA1c data in our sample [12, 24].

These findings have implications for clinical practice and may help inform healthcare professionals working with adolescents with T1D. Addressing the most adaptive coping strategies to use for specific stressors related to diabetes management may help adolescents effectively improve glycemic and psychosocial outcomes. Our findings suggest that primary and secondary control coping strategies may both be beneficial to manage psychosocial challenges related to diabetes management, and that determining the most adaptive coping strategy may depend on the controllability of the stressor. For example, if the pain associated with insulin injections or insertions of diabetes devices is a stressor for adolescents, a secondary control coping strategy such as distraction from the task may be most helpful (e.g., *I think about happy things to take my mind off the problem or how I’m feeling* [16]) [25]. Similarly, if feeling different from their peers is a stressor for adolescents, secondary control coping, or acceptance, may help them positively incorporate T1D into their identity (e.g., *I just take things as they are, I go with the flow* [16]) [26]. On the other hand, adolescents who feel like their caregivers are constantly nagging them to complete diabetes-related tasks may benefit from using primary control coping strategies, such as problem-solving, to identify more productive communication strategies (e.g., *I ask other people for help or for ideas about how to make the problem better* [16]) [27]. Finally, for managing psychosocial and glycemic challenges, providers can encourage adolescents to reduce the use of disengagement coping strategies (avoidance, denial) consistently associated with negative outcomes in adolescents with T1D and the general adolescent population.

In our sample, glycemic outcomes were associated with primary control coping strategies and disengagement coping strategies, but not secondary control coping strategies. Healthcare professionals can help adolescents distinguish the controllability of various stressors, which may promote the use of primary and secondary control coping strategies where appropriate, thus helping adolescents manage some of these diabetes-specific stressors [12]. Further research may be warranted to adapt the RSQ control-based coping strategy model to ask adolescents which coping strategies they use in response to specific diabetes stressors. This may increase our understanding of controllable versus uncontrollable stressors for adolescents with T1D.

The diverse sample of adolescent participants in this study allowed us to examine demographic differences in the use of coping strategies that could inform clinical practice and future interventions. First, we found that males reported greater use of primary and secondary control strategies than females. It is important to note that these are based on ratio scores, so it is not simply that males report using more coping strategies than females. These findings are in line with other studies of coping in adolescents with chronic illnesses [28] and suggest that females may need encouragement to use engagement coping strategies to manage diabetes-related stressors. In addition, we found that Black adolescents reported higher use of disengagement coping strategies than their peers from another race or ethnicity. These findings are consistent with previous findings from a general sample of adolescents which demonstrated that Black adolescents report higher use of disengagement coping strategies [29]. Given findings from the current study and the other studies of coping in youth with T1D linking the use of disengagement strategies to poorer psychosocial and health outcomes [30], future culturally relevant interventions may be needed to reduce the use of these strategies among Black adolescents.

The diverse population of adolescents with T1D included here is a major strength of the study. Participants were recruited from two large pediatric diabetes clinics, encompassing patient populations from urban and rural areas, resulting in a sample that is diverse in terms of race and ethnicity, socioeconomic status, and geographic location. This study is limited by only including English-speaking participants, so it may not be generalizable to all adolescent T1D populations. Additionally, all participants reported diabetes distress which may have limited the variability of distress scores. Finally, the data are cross-sectional, and therefore directionality of the observed effects cannot be determined.

Among a sample of adolescents experiencing diabetes distress, primary control coping strategies were associated with better glycemic and psychosocial outcomes, while disengagement coping strategies were associated with worse outcomes, even after adjusting for demographic and clinical factors. Secondary control coping strategies were associated with better psychosocial outcomes, but not glycemic outcomes. Future work is needed to explore the directionality of these relationships. These findings support the development and implementation of behavioral interventions that promote the use of primary and secondary control coping strategies (depending on the controllability of a stressor) and discourage the use of disengagement coping strategies for adolescents with T1D.

## Data Availability

De-identified data from this study are not available in a public archive. De-identified data from this study will be made available (as allowable according to institutional IRB standards) by emailing the corresponding author.

## References

[1] Divers J , Mayer-Davis EJ, Lawrence JM, et al. Trends in incidence of type 1 and type 2 diabetes among youths-Selected counties and Indian Reservations, United States, 2002–2015. *Morbidity and Mortality Weekly Report*. 2020;69(6):161–165. doi:10.15585/mmwr.mm6906a3 32053581 PMC7017961

[2] Lawrence JM , Divers J, Isom S, et al.; SEARCH for Diabetes in Youth Study Group. Trends in prevalence of type 1 and type 2 diabetes in children and adolescents in the US, 2001–2017. JAMA. 2021;326(8):717–727. doi:10.1001/jama.2021.11165 34427600 PMC8385600

[3] Casey BJ , Jones RM, Levita L, et al. The storm and stress of adolescence: insights from human imaging and mouse genetics. Dev Psychobiol. 2010;52(3):225–235. doi:10.1002/dev.20447 20222060 PMC2850961

[4] Ingram J , Ohan J, Bebbington K. Diabetes stigma predicts higher HbA1c levels in Australian adolescents with type 1 diabetes. Stigma Health. 2022;7(4):454–460. doi:10.1037/sah0000408.supp

[5] Malik JA , Koot HM. Explaining the adjustment of adolescents with type 1 diabetes: role of diabetes-specific and psychosocial factors. Diabetes Care. 2009;32(5):774–779. doi:10.2337/dc08-1306 19196897 PMC2671093

[6] Demeterco-Berggren C , Ebekozien O, Noor N, et al. Factors associated with achieving target A1C in children and adolescents with type 1 diabetes: findings from the T1D exchange quality improvement collaborative. Clin Diabetes. 2023;41(1):68–75. doi:10.2337/cd22-0073 PMC9845079 36714245

[7] Hilliard ME , Herzer M, Dolan LM, Hood KK. Psychological screening in adolescents with type 1 diabetes predicts outcomes one year later. Diabetes Res Clin Pract. 2011;94(1):39–44. doi:10.1016/j.diabres.2011.05.027 21665313 PMC3192912

[8] Inverso H , Lestourgeon LM, Parmar A, et al. Demographic and glycemic factors linked with diabetes distress in teens with type 1 diabetes. J Pediatr Psychol. 2022;47(9):1081–1089. doi:10.1093/jpepsy/jsac049 35656859 PMC9801711

[9] Agarwal S , Kanapka LG, Raymond JK, et al. Racial-ethnic inequity in young adults with type 1 diabetes. J Clin Endocrinol Metab. 2020;105(8):e2960–e2969. doi:10.1210/clinem/dgaa236 32382736 PMC7457963

[10] Hagger V , Hendrieckx C, Cameron F, Pouwer F, Skinner TC, Speight J. Diabetes distress is more strongly associated with HbA1c than depressive symptoms in adolescents with type 1 diabetes: results from Diabetes MILES Youth—Australia. Pediatr Diabetes. 2018;19(4):840–847. doi:10.1111/pedi.12641 29383803

[11] Sturt J , Dennick K, Due-Christensen M, McCarthy K. The detection and management of diabetes distress in people with type 1 diabetes. Curr Diab Rep. 2015;15(11):101. doi:10.1007/s11892-015-0660-z 26411924

[12] Jaser SS , Patel N, Xu M, Tamborlane WV, Grey M. Stress and coping predicts adjustment and glycemic control in adolescents with type 1 diabetes. Ann Behav Med. 2017;51(1):30–38. doi:10.1007/s12160-016-9825-5 27496164 PMC5253083

[13] Yaribeygi H , Panahi Y, Sahraei H, Johnston TP, Sahebkar A. The impact of stress on body function: a review. EXCLI J. 2017;16:1057–1072. doi:10.17179/excli2017-480 28900385 PMC5579396

[14] Hilliard ME , Hagger V, Hendrieckx C, et al. Strengths, risk factors, and resilient outcomes in adolescents with type 1 diabetes: results from diabetes MILES Youth-Australia. Diabetes Care. 2017;40(7):849–855. doi:10.2337/dc16-2688 28446529 PMC5481988

[15] Compas BE , Jaser SS, Bettis AH, et al. Coping, emotion regulation, and psychopathology in childhood and adolescence: a meta-analysis and narrative review. Psychol Bull. 2017;143(9):939–991. doi:10.1037/bul0000110 28616996 PMC7310319

[16] Connor-Smith JK , Compas BE, Wadsworth ME, Thomsen AH, Saltzman H. Responses to stress in adolescence: measurement of coping and involuntary stress responses. J Consult Clin Psychol. 2000;68(6):976–992. doi:10.1037/0022-006X.68.6.976 11142550

[17] Hilliard ME , Iturralde E, Weissberg-Benchell J, Hood KK. The Diabetes Strengths and Resilience Measure for Adolescents with Type 1 Diabetes (DSTAR-Teen): validation of a new, brief self-report measure. J Pediatr Psychol. 2017;42(9):995–1005. doi:10.1093/jpepsy/jsx086 28549160 PMC6075601

[18] Jaser SS , Datye K, Morrow T, et al. THR1VE! Positive psychology intervention to treat diabetes distress in teens with type 1 diabetes: rationale and trial design. Contemp Clin Trials. 2020;96:106086. doi:10.1016/j.cct.2020.106086 32682996 PMC7494522

[19] Shapiro JB , Vesco AT, Weil LEG, Evans MA, Hood KK, Weissberg-Benchell J. Psychometric properties of the problem areas in diabetes: teen and parent of teen versions. J Pediatr Psychol. 2018;43(5):561–571. doi:10.1093/jpepsy/jsx146 29267939 PMC6454555

[20] Hilliard ME , Minard CG, Marrero DG, et al. Assessing health-related quality of life in children and adolescents with diabetes: development and psychometrics of the Type 1 Diabetes and Life (T1DAL) measures. J Pediatr Psychol. 2020;45(3):328–339. doi:10.1093/jpepsy/jsz083 31665389 PMC7850123

[21] Elsayed NA , Aleppo G, Aroda VR, et al.; on behalf of the American Diabetes Association. 6. Glycemic targets: standards of care in diabetes—2023. Diabetes Care. 2023;46(Suppl 1):S97–S110. doi:10.2337/dc23-S006 36507646 PMC9810469

[22] Jaser SS , White LE. Coping and resilience in adolescents with type 1 diabetes. Child Care Health Dev. 2011;37(3):335–342. doi:10.1111/j.1365-2214.2010.01184.x 21143270 PMC3134245

[23] Yi-Frazier JP , Yaptangco M, Semana S, et al. The association of personal resilience with stress, coping, and diabetes outcomes in adolescents with type 1 diabetes: variable- and person-focused approaches. J Health Psychol. 2015;20(9):1196–1206. doi:10.1177/1359105313509846 24271691 PMC5106185

[24] Luyckx K , Seiffge-Krenke I, Hampson SE. Glycemic control, coping, and internalizing and externalizing symptoms in adolescents with type 1 diabetes: a cross-lagged longitudinal approach. Diabetes Care. 2010;33(7):1424–1429. doi:10.2337/dc09-2017 20357383 PMC2890333

[25] Taraban L , Wasserman R, Cao VT, et al. Diabetes-related worries and coping among youth and young adults with type 1 diabetes. J Pediatr Psychol. 2022;47(10):1145–1155. doi:10.1093/jpepsy/jsac055 35773974 PMC9582784

[26] Commissariat PV , Volkening LK, Weinzimer SA, Dassau E, Laffel LM. Assessing incorporation of type 1 diabetes into identity: validation of the Accepting Diabetes and Personal Treatment (ADAPT) Survey in teens and young adults. Can J Diabetes. 2023;47(1):66–72. doi:10.1016/j.jcjd.2022.08.007 36184368 PMC10096441

[27] Whittemore R , Zincavage RM, Jaser SS, et al. Development of an eHealth program for parents of adolescents with type 1 diabetes. Diabetes Educ. 2018;44(1):72–82. doi:10.1177/0145721717748606 29262747 PMC6330708

[28] Al-Yateem N , Arsyad Subu M, Al-Shujairi A, et al. Coping among adolescents with long-term health conditions: a mixed-methods study. Br J Nurs. 2020;29(13):762–769. 32649257 10.12968/bjon.2020.29.13.762

[29] McWood LM , Erath SA, El-Sheikh M. Longitudinal associations between coping and peer victimization: moderation by gender and initial peer victimization. Soc Dev. 2023;32(1):117–134. doi:10.1111/sode.12623 36874168 PMC9983818

[30] Iturralde E , Weissberg-Benchell J, Hood KK. Avoidant coping and diabetes-related distress: pathways to adolescents’ type 1 diabetes outcomes. Health Psychol. 2017;36(3):236–244. doi:10.1037/hea0000445 27808528 PMC7302678

